# Fibrous Pseudotumor of Tunica Albuginea Testis Mimicking Testicular Neoplasm in a Young Man

**DOI:** 10.1155/2018/9315864

**Published:** 2018-06-20

**Authors:** Syed Muhammad Nazim, Ayesha Nusrat, Zehra Kazmi

**Affiliations:** ^1^Urology Section, Department of Surgery, Aga Khan University, Karachi, Pakistan; ^2^Histopathology Section, Department of Pathology, Aga Khan University, Karachi, Pakistan

## Abstract

Intrascrotal lesions are common findings with a majority occurring in paratesticular tissue. Fibrous pseudotumors are rare, benign lesions of the testicular tunics and present with mass lesion(s) in the scrotum. Preoperative clinical and radiological diagnosis is challenging. We report a case of a 34-year-old man who presented with a 3-year history of left testicular swelling and was advised left radical orchidectomy by another surgeon. Physical examination revealed a firm, nontender mass attached to the lower pole of the testis. Testicular tumor markers were all negative, and ultrasound scan showed a relatively hypoechoic lesion closely associated with the left testis and suspicious for neoplastic process. The patient underwent a testicular sparing surgery. An intraoperative frozen section biopsy confirmed the lesion to be benign and this was reported on permanent section to be fibrous pseudotumor of the tunica albuginea. We also present the clinical, sonographic, and histopathological findings of this condition along with the literature review.

## 1. Introduction

Intrascrotal lesions are common findings in the male population with a majority occurring in paratesticular tissue. Contrary to testicular lesions which are 95% malignant, most paratesticular lesions are benign [1, 2]. Fibrous pseudotumors are rare, non-neoplastic benign lesions of the testicular tunics and present with single or more nodular mass lesions in the scrotum [3, 4]. The peak incidence is reported between 2nd and 4th decades of life.

Comprising only 6% of paratesticular tumors, these lesions pose a diagnostic and therapeutic dilemma due to their confusion with malignant lesions [3].

Preoperative diagnosis of fibrous pseudotumors is challenging both clinically and radiologically [2]. Many of these lesions have been operated with radical orchidectomy despite the benign nature of the disease [2, 4]. Instead of aggressive surgeries, testicular sparing surgery should be done to preserve the fertility potential of younger patients.

We report case of a young man who came for a second opinion at our clinic for left scrotal swelling for which he was advised a left radical orchidectomy by another surgeon. Surgeons should be aware of this rare condition and must do a careful evaluation to prevent unnecessary removal of the testis.

## 2. Case Presentation

A 34-year-old man, father of 2 kids, presented to urology outpatient clinic complaining of swelling in the left testis for the last 3 years. The swelling gradually increased in size and was not associated with any pain or fever. The patient also denied prior history of any trauma, infections, or scrotal surgeries. The past medical and surgical history was otherwise unremarkable.

Physical examination revealed a circumcised penis with bilateral developed hemiscrotum and normally descended testes. The right testis was normal, and the left testis had a firm, nontender 2.5 × 2.0 cm smooth mass attached near its lower pole with an associated lax hydrocele.

### 2.1. Investigations

**Figure 1:**
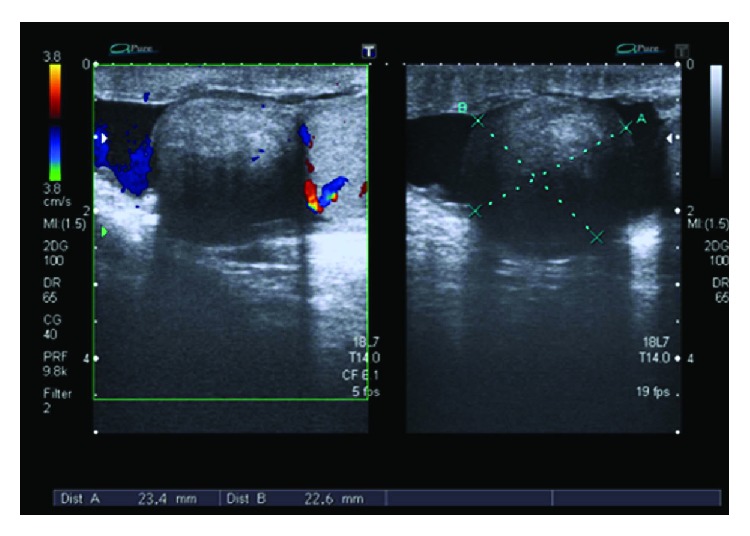
Gray scale and Doppler ultrasound scans showing well-circumscribed lesion separated from the testis and relatively hypoechoic compared to testicular parenchyma with no significant vascularity on color Doppler.

The baseline hematological and biochemistry workup was normal. Serum markers for germ cell tumor of the testes were all normal with serum lactate dehydrogenase (LDH) 275 IU/l (*N* = 208–378), alpha feto protein (*α*FP) 4.0 IU/ml (*N* ≤ 6.7), and beta human chorionic gonadotropin (*β*HCG) <2.0 mIU/ml (*N* < 10). An ultrasound scan was done which showed a 23.4 mm × 22.6 mm well-circumscribed lesion closely associated with the left testis. On color Doppler, no significant vascularity was observed (Figure 1). The radiologist could not definitely determine the nature of the lesion, and it was labeled as suspicious for neoplastic process. An ultrasound of the abdomen did not reveal any evidence of lymphadenopathy.

## 3. Differential Diagnosis

The differential diagnosis includes testicular germ cell tumor which is common in the same age group. However, in our patient, the tumor markers were all normal and the lesion was slow growing over a period of 3 years. There was no risk factor for testicular tumor, and family history was also negative. The patient also denied any constitutional symptoms. Another differential diagnosis is tumor of gonadal stromal origin which is rare and has both fibromatous and sex cord components [5]. Its exclusion is important as it is malignant.

Other differential diagnoses include other paratesticular lesions such as spermatocele, hydrocele, varicocele, polyorchidism, intratesticular simple cyst, and tumors of the spermatic cord such as lipoma and leiomyoma. The mass in our patient was well circumscribed and though attached to the testicular capsule, its echogenicity was different from the left testis. It was a noncystic, solid lesion, and there were no dilated peritesticular veins. There was an associated hydrocele.

### 3.1. Surgery

After a detailed discussion with the patient and family and obtaining an informed consent, a decision was made for left inguinal exploration with intraoperative frozen section biopsy of the lesion and to proceed accordingly.

**Figure 2:**
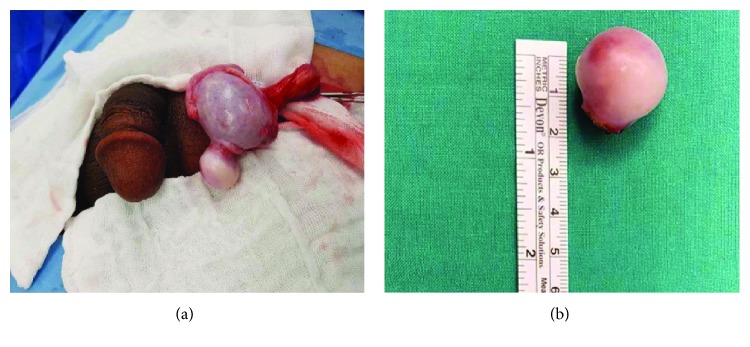
Intraoperative picture of the left testis with an attached mass (a). Gross appearance of the excised lesion (b).

A groin skin crease incision was made, spermatic cord was mobilized in the inguinal canal, and the left testis with its coverings was delivered. There was mild hydrocele with 15–20 ml of amber-colored fluid, and a 2.5 × 2.5 cm smooth mass was found attached to the left testis (Figure 2). Biopsies were taken for intraoperative frozen section which showed benign inflammatory fibrotic tissue, so the lesion was completely excised and the tunica albuginea was repaired. Hence, a testicular sparing surgery was performed.

### 3.2. Outcome and Follow-Up

**Figure 3:**
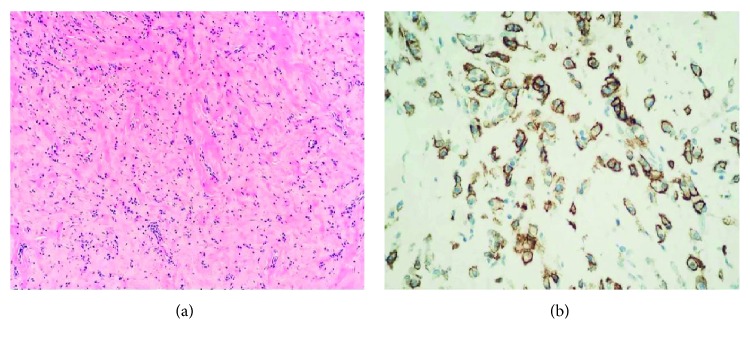
(Hematoxylin and eosin, original magnification ×100.) Densely hyalinized fibrous tissue with scattered aggregates of chronic inflammatory cells (a) and high-power view (original magnification ×400). Scattered plasma cells highlighted on immunohistochemical stain CD138 (b).

The patient made an uneventful recovery. The final histopathology report showed that on gross examination, the tumor consisted of a grey-white capsulated nodular tissue, firm on the cut surface. On microscopy, there was a thick fibrous-capsule-covered hyalinized fibrous tissue with scattered aggregates of lymphocytes and plasma cells (Figure 3). On immunohistochemical stain, ALK protein, ASMA, and desmin were negative in these cells. The case was discussed in our tumor board meeting, and subsequently, the diagnosis was communicated to the patient. On the last follow-up at 6 months, the patient was doing well with no recurrence of the lesion.

## 4. Discussion

Fibrous pseudotumors represent an uncommon clinical diagnosis and were first reported by Balloch in 1904 [1]. They are classified as benign paratesticular tumors and commonly involve the testicular tunics, mainly tunica vaginalis (75%), but are also associated with tunica albuginea, epididymis, and spermatic cord [6, 7]. Following adenomatoid tumor and spermatic cord lipoma, they represent the 3rd commonest paratesticular mass [8]. Multiple names have been applied for these lesions including inflammatory pseudotumor, chronic proliferative periorchitis, proliferative funiculitis, fibromatous periorchitis, fibrous mesothelioma, benign fibrous paratesticular tumor, and reactive periorchitis [6, 7].

Jones et al. [5] have proposed 2 neoplastic forms of benign fibrous tumor of the testis and its adnexa: fibroma of gonadal stromal origin and fibroma of testicular tunics. The etiology of fibrous pseudotumors is unknown, but the pathogenesis is thought to be due to benign the fibro-inflammatory reaction in response to chronic inflammation [9]. These lesions are thought to originate from fibroblasts and myofibroblasts. In some cases, a history of prior surgery or trauma and association of infection or inflammatory hydrocele with this condition also supports its reactive nature.

Williams and Banerjee [10] studied 114 paratesticular tumors and found only 7 cases of fibrous pseudotumors of which 6 were removed by orchidectomy. These tumors have peak incidence in the 3rd decade of life but can occur at any age [9]. Majority of patients present with a painless scrotal swelling [3, 11], and on clinical examination, these lesions are palpated as single or multiple painless, firm masses ranging from 0.5 to 8 cm [1].

Occasionally, these nodules detach from the tunical surface giving rise to scrotal pearls and floaters in tunical space [1]. They appear as hypoechoic or hyperechoic solid mass on an ultrasound (U/S) scan attached to or closely associated with the capsule of the testis [4]. A hydrocele is often associated. A dense fibrotic tissue within the lesion with associated calcification can also give rise to acoustic shadowing [12]. A color Doppler ultrasound may reveal mild vascularity within the lesion.

On magnetic resonance imaging (MRI), the lesions have intermediate-to-low signal intensity on T1W sequence (similar to that of the testis) whereas they have low signal intensity on T2W images [12]. There is no or minimal enhancement on postcontrast images. Both U/S and MRI, however, have limitations in that they cannot determine the nature of the lesions whether they are benign or malignant.

On gross appearance, they are well circumscribed and white-tan or yellowish in color and can be bulging or whorled on the cut section [5]. On microscopy, there is multinodular or diffuse paucicellular fibroblast proliferation with abundant hyalinized collagen [5]. Dispersedly distributed inflammatory cell infiltrate comprising of plasma cells, lymphocytes, and occasional eosinophils can also be seen [5, 11]. Immunohistochemical staining of fibrous pseudotumors is positive for vimentin, smooth-muscle-specific actin and common muscle actin [7, 11]. These pseudotumors are negative for S-100, keratin, and desmin [5].

The treatment of choice for paratesticular fibrous pseudotumors is surgical excision. Intraoperative frozen section biopsy is recommended when the testis is involved with the tumor [13] and can prevent orchidectomy in a young patient. In case of high suspicion of malignancy or where fibrotic tissue diffusely occupies and encases the testicular tissue (fibromatous periorchitis), orchidectomy can be selected as a surgical procedure. The prognosis of fibrous pseudotumors is excellent, and recurrence after complete excision is extremely rare [2].

## Additional Points

*Take-Home Messages*. (i) Fibrous pseudotumors are rare cause of scrotal swelling which can be confused with malignant lesions. (ii) Preoperative diagnosis of these lesions is challenging, and both radiologists and surgeons should be aware of this condition as unfamiliarity with fibrous pseudotumors may result in orchidectomy. (iii) Intraoperative frozen section assessment is helpful to establish the correct diagnosis and is essential for testicular sparing procedures.
